# Morphological changes in the trabecular meshwork and Schlemm’s canal after treatment with topical intraocular pressure-lowering agents

**DOI:** 10.1038/s41598-021-97746-x

**Published:** 2021-09-13

**Authors:** Ji-Hye Park, Hyun Woo Chung, Eun Gyu Yoon, Min Jung Ji, Chungkwon Yoo, Yong Yeon Kim

**Affiliations:** 1 Department of Ophthalmology, Korea University Ansan Hospital, 123 Jeokgeum-ro, Danwon-gu, Ansan-si, Gyeonggi-do 15355 South Korea; 2 Seoul Best Eye Clinic, Seoul, South Korea

**Keywords:** Optic nerve diseases, Anatomy, Tomography

## Abstract

Glaucoma treatment is usually initiated with topical medication that lowers the intraocular pressure (IOP) by reducing the aqueous production, enhancing the aqueous outflow, or both. However, the effect of topical IOP-lowering medications on the microstructures of the aqueous outflow pathway are relatively unknown. In this retrospective, observational study, 56 treatment-naïve patients with primary open-angle glaucoma were enrolled. Images of the nasal and temporal corneoscleral limbus were obtained using anterior segment optical coherence tomography (AS-OCT). The conjunctival vessels and iris anatomy were used as landmarks to select the same limbal area scan, and the trabecular meshwork (TM) width, TM thickness, and Schlemm’s canal (SC) area were measured before and after using the IOP-lowering agents for 3 months. Among the 56 patients enrolled, 33 patients used prostaglandin (PG) analogues, and 23 patients used dorzolamide/timolol fixed combination (DTFC). After 3 months of DTFC usage, the TM width, TM thickness, and SC area did not show significant changes in either the nasal or temporal sectors. Conversely, after prostaglandin analog usage, the TM thickness significantly increased, and the SC area significantly decreased (all *P* < 0.01). These findings warrant a deeper investigation into their relationship to aqueous outflow through the conventional and unconventional outflow pathways after treatment with PG analogues.

## Introduction

Glaucoma is an optic neuropathy characterized by progressive structural and functional damage that causes irreversible blindness worldwide^1,2^. Among various risk factors, increased intraocular pressure (IOP) is the most important risk factor for glaucoma development and progression, and IOP reduction is the only proven method to prevent or slow the progression of the disease^3^. The main cause of IOP increase is not an overproduction of aqueous humor but an increased resistance to aqueous outflow. The aqueous humor flows out of the eye through two main outflow pathways^4,5^. Most aqueous humor is drained through a system consisting of the trabecular meshwork (TM), Schlemm’s canal (SC), intrascleral collector channels (CCs), and episcleral and conjunctival veins. This outflow pathway, referred to as the conventional or trabecular outflow pathway, accounts for approximately 70% to 95% of the aqueous humor outflow, and the major resistance sites are localized to the juxtacanalicular portion of the TM and inner wall of SC^4^. In the unconventional or uveoscleral outflow, the aqueous humor leaves the eye through the iris root, between the ciliary muscle bundles, and then through the suprachoroidal and scleral tissues^5^. The uveoscleral outflow pathway is responsible for 5% to 30% of the aqueous humor outflow, and the main flow-limiting part is formed by the resistance of the muscle bundles and connective tissue of the ciliary body.

Technological advances in anterior segment imaging methods have enabled a noninvasive, in vivo investigation of the aqueous outflow pathway^6–8^. Previous studies have scanned the corneoscleral limbus using anterior segment optical coherence tomography (OCT) and shown the usefulness of OCT in visualizing both the pre- and post-trabecular outflow pathways^6–10^. The microstructure morphology of the outflow pathway obtained by OCT corresponded well with ex vivo histologic study findings from enucleated eyes^11,12^. Li and coworkers^8^ used enhanced depth imaging (EDI) OCT to examine in vivo SC and CC microstructures in normal subjects and reported excellent repeatability and reproducibility of visualizing SC and the CCs. In the report, the SC area varied considerably among subjects and tended to be larger in regions with more CCs. Hong et al.^10^ evaluated the in vivo features of SC in patients with primary open-angle glaucoma (POAG) and compared them with those in normal subjects. They revealed that the SC area of patients with POAG was significantly smaller than that of normal subjects, and there was a significant relationship between the IOP and SC area. Outflow pathway information obtained on anterior segment OCT may play an important role in elucidating the mechanism of increased IOP and consequently the pathophysiology of glaucoma development.

Currently, glaucoma treatment is usually initiated with topical medication that lowers the IOP by reducing the aqueous production, enhancing the aqueous outflow, or both^13^. Although there are some studies reporting the morphologic changes in the TM and SC after using topical medication or after incisional surgery^14–18^, there is no in vivo study reporting the relatively long-term effect of topical IOP-lowering medications on the microstructures of the aqueous outflow pathway. Therefore, we evaluated the alterations in the TM and SC after using topical IOP-lowering medications for 3 months in treatment-naïve patients with POAG.

## Results

A total of 63 subjects were consecutively enrolled herein. Among them, seven subjects were excluded because of the following reasons: change in medication owing to ocular side effects (three patients), poor image quality (three patients), and loss of follow-up (one patient). Thus, 56 treatment-naïve patients with glaucoma (mean age: 50.41 ± 13.19 years) were included in the analysis (Table 1). Thirty-three and twenty-three patients were prescribed prostaglandin (PG) analogues and dorzolamide/timolol fixed combination (DTFC), respectively. Among the PG group, 16, 15, and 2 patients used latanoprost, bimatoprost, and tafluprost, respectively. Although the patients in the PG group were younger than those in the DTFC group, there was no significant difference in the untreated IOP, axial length, visual field (VF) parameters, and retinal nerve fiber layer (RNFL) thickness between them. The measurements of the TM and SC microstructure showed good inter-observer agreement (Supplementary Table 1, intraclass correlation coefficient, ICC, 0.912 ~ 0.982).

**Table 1:** Baseline Characteristics of the Subjects.

	Total subjects (n = 56)	Prostaglandins (n = 33)	DTFC (n = 23)	*P* value*
Age (y)	50.41 ± 13.19	53.67 ± 13.20	45.74 ± 11.96	0.026^†^
Untreated IOP (mmHg)	17.6 ± 3.6	17.6 ± 3.3	17.6 ± 4.1	0.644
Axial length (mm)	24.45 ± 1.45	24.45 ± 1.30	24.44 ± 1.66	0.980^†^
Spherical equivalent (D)	− 1.79 ± 2.65	− 2.08 ± 2.64	− 1.39 ± 2.68	0.233
CCT (μm)	526.41 ± 38.42	529.58 ± 35.82	521.87 ± 42.27	0.571
ACD (mm)	3.41 ± 0.41	3.45 ± 0.46	3.36 ± 0.33	0.392^†^
MD (dB)	− 4.88 ± 4.29	− 4.94 ± 4.30	− 4.80 ± 4.36	0.874
PSD (dB)	4.94 ± 3.86	4.60 ± 3.35	5.42 ± 4.54	0.907
VFI (%)	89.62 ± 11.82	90.33 ± 11.14	88.61 ± 12.92	0.967
RNFL thickness (μm)	82.91 ± 15.64	84.39 ± 12.82	80.78 ± 19.10	0.400^†^
TM width (μm)				
Nasal sector	507.02 ± 68.85	517.32 ± 74.96	493.13 ± 58.36	0.431
Temporal sector	512.08 ± 59.62	499.55 ± 61.29	530.57 ± 53.17	0.099
TM thickness (μm)				
Nasal sector	89.67 ± 16.20	88.46 ± 15.14	91.22 ± 17.74	0.901
Temporal sector	89.53 ± 15.71	91.55 ± 14.84	86.69 ± 16.81	0.170
SC area (μm^2^)				
Nasal sector	4696.46 ± 1446.72	4883.57 ± 1407.05	4444.26 ± 1492.21	0.252
Temporal sector	4561.72 ± 1490.30	4665.07 ± 1624.13	4409.16 ± 1290.68	0.569

*Mann–Whitney U test, ^†^Independent t-test.

DTFC, dorzolamide/timolol fixed combination; IOP, intraocular pressure; CCT, central corneal thickness; ACD, anterior chamber depth; MD, mean deviation; PSD, pattern standard deviation; VFI, visual field index; RNFL, retinal nerve fiber layer; TM, trabecular meshwork; SC, Schlemm’s canal.

The IOP, central corneal thickness (CCT), TM width, TM thickness, and SC area before and after IOP-lowering medication usage are demonstrated in Table 2. When the changes were evaluated individually for each medication, the TM width, TM thickness, and SC area did not show significant changes in the DTFC group (*P* > 0.05). However, the TM thickness significantly increased when PG analogues were used (nasal sector, *P* < 0.001; temporal sector, *P* = 0.007). Moreover, the SC area significantly decreased in the PG group (both sectors, *P* = 0.001). When the correlation between the amount of IOP change and the amount of TM width, TM thickness, or SC area change were evaluated, there was a negative correlation found between the IOP change and TM thickness change (Spearman’s rho =—0.415, *P* < 0.001, Table 3) in the PG group, indicating that a large amount of IOP reduction was associated with a significant increase in the TM thickness.

**Table 2:** Comparison of the TM Width, TM thickness, and SC Area Before and After Topical Medication Usage.

	Prostaglandin (n = 33)			DTFC (n = 23)		
	Before	After 3 months	*P* value*	Before	After 3 months	*P* value*
IOP (mmHg)	17.6 ± 3.3	13.6 ± 2.8	< 0.001	17.6 ± 4.1	15.3 ± 2.9	0.003
CCT (μm)	528.50 ± 35.84	519.91 ± 34.42	< 0.001	521.82 ± 43.27	527.64 ± 41.81	0.018
TM width (μm)						
Nasal sector	517.32 ± 74.96	515.16 ± 69.18	0.590	493.13 ± 58.36	489.83 ± 54.57	0.592
Temporal sector	499.55 ± 61.29	501.45 ± 68.49	0.845	530.57 ± 53.17	532.71 ± 58.09	0.394
TM thickness (μm)						
Nasal sector	87.98 ± 14.52	99.07 ± 18.71	< 0.001	93.90 ± 19.88	96.97 ± 18.06	0.375
Temporal sector	90.36 ± 15.66	97.23 ± 19.61	0.007	87.05 ± 17.34	90.72 ± 17.20	0.211
SC area (μm^2^)						
Nasal sector	4883.57 ± 1407.05	4319.59 ± 1135.06	0.001	4444.26 ± 1492.21	4516.82 ± 1522.34	0.879
Temporal sector	4665.07 ± 1624.13	4076.50 ± 1333.79	0.001	4409.16 ± 1290.68	4269.32 ± 1025.46	0.543

*Wilcoxon signed-rank test.

TM, trabecular meshwork; SC, Schlemm’s canal; DTFC, dorzolamide/timolol fixed combination; IOP, intraocular pressure; CCT, central corneal thickness.

**Table 3:** Correlation coefficient between IOP Changes and TM Width, TM Thickness, and SC Area Measurements.

	Baseline						Changes					
	TM width		TM thickness		SC area		TM width		TM thickness		SC area	
	Nasal sector	Temporal sector	Nasal sector	Temporal sector	Nasal sector	Temporal sector	Nasal sector	Temporal sector	Nasal sector	Temporal sector	Nasal sector	Temporal sector
*△IOP*												
PG	0.157	0.058	− 0.161	− 0.206	− 0.280	− 0.028	− 0.167	− 0.152	− 0.415*	0.058	0.022	0.200
DTFC	0.157	0.439*	0.017	− 0.229	− 0.020	0.244	0.126	0.389	− 0.209	0.281	− 0.143	− 0.258

Spearman’s correlation test, *P < 0.01.

IOP, intraocular pressure; TM, trabecular meshwork; SC, Schlemm’s canal; PG, prostaglandin; DTFC, dorzolamide/timolol fixed combination.

**Figure 1:**
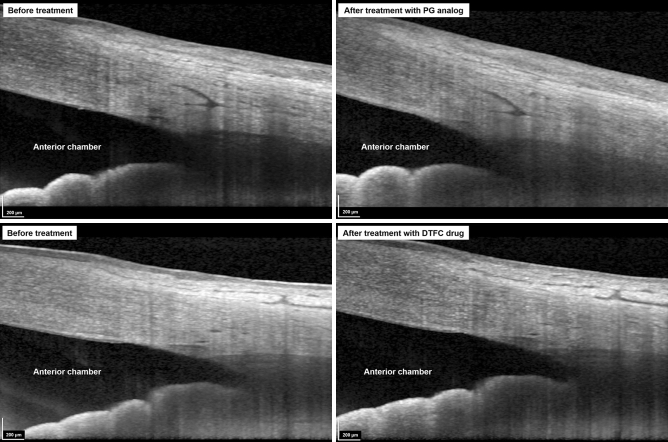
Enhanced depth imaging optical coherence tomography B-scans before (Left) and after (Right) administration of topical intraocular pressure-lowering agents. (Top) Trabecular meshwork thickness increase and Schlemm’s canal area reduction are observed after usage of prostaglandin analog for 3 months. (Bottom) The Schlemm’s canal area did not show significant changes after usage of dorzolamide/timolol fixed combination (DTFC) drug.

In the subgroup analysis, the PG group was subdivided into two groups according to the IOP reduction amount: group 1 (△IOP of < 4 mmHg, small IOP reduction group) and group 2 (△IOP of ≥ 4 mmHg, large IOP reduction group). As the mean IOP reduction in the PG group was—3.9 ± 2.5 mmHg, we selected 4 mmHg as the criterion for subdividing the group. The ocular variables were compared using the Mann–Whitney U test between them (Supplementary Table 2). Although the baseline TM and SC parameters were not significantly different between them, the large IOP reduction group showed greater TM thickness changes in the nasal sector than did the small IOP reduction group (13.85 ± 13.02 μm vs. 5.33 ± 12.32 μm, *P* = 0.027). Figure 1 shows representative cases of TM thickness and SC area changes before and after using each medication.

## Discussion

In the present study, observations of naïve patients with POAG treated with PG analogues displayed increased TM thickness and reduced SC area. Additionally, IOP reduction was also present along with the increase in TM thickness in the PG group. However, the TM width, TM thickness, and SC area did not show significant changes in the DTFC group. To the best of our knowledge, this is the first study to evaluate the microstructural alterations in the TM and SC after topical IOP-lowering medication usage for 3 months in treatment-naïve patients with POAG.

The main resistance sites are the juxtacanalicular connective tissue of the TM and inner wall of SC in the conventional outflow pathway. The TM and SC microstructure is important in aqueous outflow and consequently in IOP regulation. Further, histologic studies have revealed that the outflow capacity and dimension of outflow pathway sites have a strong correlation^19,20^. Allingham et al.^20^ reported that the eyes with POAG had significantly smaller SC cross-sectional area and perimeter than the normal eyes. They suggested that SC dimension reduction may play an important role in decreasing outflow facilities in the eyes with POAG. Furthermore, Rohen et al.^21^ showed thickened trabeculae and increased amounts of plaque-material deposited within the TM cribriform layer in patients with POAG.

With advances in imaging technology, recent studies have utilized OCT to investigate the association between the microstructure of the outflow pathway and glaucoma status^10,22^ or IOP level^20,24,25^. Hong et al.^10^ showed that the SC area in patients with POAG was significantly smaller than that in normal subjects, and there was a significant relationship between the IOP and SC area. Additionally, Masis and coworkers^22^ reported that patients with primary angle-closure glaucoma (PACG) had shorter TM widths than patients with POAG. Qi and colleagues^23^ reported the morphological features of SC and the TM in highly myopic eyes with early IOP elevation after cataract surgery. They found a smaller vertical SC diameter and a thinner TM as two risk factors for early IOP elevation after cataract surgery. They suggested that these anatomical features indicate a low capability to deal with aqueous drainage during the early postoperative period. Furthermore, several studies have reported that glaucoma treatments, such as medications^16^, laser treatment^26^, and surgery^14^, can change the TM and SC microstructure. Previously, Chen et al.^16^ evaluated the effect of travoprost 0.004% on the SC area using Fourier-domain OCT (FD-OCT) in 12 healthy subjects. They instilled one drop of travoprost or placebo eye drops in the morning, and the IOP measurements and FD-OCT scans were compared before and after eye drop instillation. While the SC area remained stable in the placebo group, the SC lumen expanded obviously, and the IOP decreased in the travoprost-treated group; the expansion effect was maintained up to 60 h after administration. However, this expansion was reversed to the baseline from 72 h. In line with this finding, it has been reported that IOP reduction leads to SC area expansion and vice versa^20,24,25^. Based on microsurgical and perfusion study, Johnstone and Grant^24^ reported that the diameter of the SC lumen was IOP-dependent and reached a minimum at high pressures but was enlarged under low pressure. In another study, Chen and colleagues^25^ showed the link between the collapse of SC and decrease in outflow facility with acute IOP increase. Further, Hong et al.^14^ reported that the degree of SC expansion was related to the extent of IOP decrease after trabeculectomy in patients with glaucoma. However, in contrast to previous findings, the SC area significantly decreased with IOP reduction after PG analog usage in this study. Such inconsistent results between prior studies and ours may be attributed to several reasons.

First, most previous studies have dramatically increased or decreased the IOP by compressing the eyeball with an ophthalmodynamometer, perfusing the eye experimentally, or performing incisional surgery and evaluated the outflow changes. It is assumed that experimental changes are different from physiological changes caused by drug use. Second, the association between IOP elevation and SC area reduction, and vice versa, might not be found in all patients. Johnson and Matsumoto^15^ showed that in the eyes with POAG that have received filtration surgeries, low IOP was correlated with the small SC area. Third, PG analog usage for 3 months may yield structural changes different from those with short-term usage of the drug. PG analogues are known to improve the uveoscleral aqueous outflow through ciliary muscle relaxation in the short term and through extracellular matrix (ECM) remodeling in the long term^27^. Thus, the structural changes may show different patterns depending on the time of observation. Consistent with our assumption, Richter et al.^28^ showed the collapse of SC in monkey eyes treated with bimatoprost for 1 year and noted differences in ciliary muscle (CM) and TM morphologies between the eyes treated with PGs at the short (up to 5 days) and long (1 year) terms.

In terms of TM changes, the TM thickness significantly increased in the PG-treated eyes, while the TM width did not significantly change. Similar to our results, in a previous study investigating long-term changes in the anterior segment of primate eyes treated for 1 year with PG agonists, most eyes showed widened cribriform region and nearly unchanged corneoscleral trabecular lamellae^28^. There is a possibility that the PG agonists affected the ion channels of the aqueous humor outflow pathway and caused changes in the TM shape^29–31^. Alternatively, the ECM alterations of scleral spur induced by PG use may have affected the morphological changes of TM (beam + empty space) recognized by AS-OCT. Additionally, IOP reduction was observed with increasing TM thickness (beams and empty space). However, the reason for the regional difference in the observations between IOP decrease and TM thickness increase observed mainly in the nasal sector, needs to be explored. Although we have no clear explanation for this finding, a possible explanation is that the nasal and temporal sectors may contribute differently to the conventional outflow. Previous studies have reported that the SC area and SC diameter were larger in the nasal than in the temporal region^6,8,32^. Furthermore, Li and coworkers^8^ reported that there were more CCs nasally than temporally. Similarly, although the difference was not significant, the SC area was greater in the nasal than in the temporal region in our study. These findings may represent the preferential nasal drainage of the aqueous humor.

In this study, DTFC did not induce microstructural alterations in the TM and SC. Dorzolamide and timolol are well-known drugs that decrease the IOP by suppressing aqueous humor production. Previous studies have suggested that hyposecretion of aqueous humor may cause underperfusion of the conventional outflow and lead to TM damage^33,34^. However, other studies reported completely opposite results; some reported no significant effect on outflow facility^35,36^, while others reported increased facility after timolol use^37,38^. Such inconsistent outcomes among the past studies may be attributed to the differences in the methodology, patient characteristics, or treatment duration. As this study did not evaluate the outflow facility, it cannot be concluded that the unchanged structures imply no change in the outflow facility. Further evaluation is needed to address the effect of medications that reduce aqueous production on outflow facilities in patients with glaucoma.

Our study has some limitations. First, the sample size was relatively small, and an uneven number of subjects were enrolled in the two groups. The small sample size may have masked the significant changes of outflow structures in DTFC group. Therefore, further studies with a large number of patients may provide a more accurate understanding of the effects of glaucoma medications on the aqueous outflow system. Additionally, only subjects whose SC and TM can be delineated were included, which might have introduced a selection bias. Second, only the relatively short-term effects of IOP-lowering medications on the TM and SC microstructures were evaluated. Previous studies have reported that the IOP-lowering effect may be different according to the duration of medication use in patients with glaucoma^37,38^. However, previous histologic studies have suggested that PG analogues induce ECM remodeling within just 3 days after drug instillation^39,40^. Therefore, further studies investigating the short- and long-term usage of topical medications are needed to elucidate the precise effects of IOP-lowering agents on aqueous outflow pathway microstructure. Third, we evaluated only the nasal and temporal meridians, which may not represent the entire aqueous outflow pathway. In addition, we cannot explain the segmental outflow pattern or its changes in this study. Finally, TM thickness and SC area changes might not be directly related to IOP reduction. However, the strength of this study is that it is the first study to explore the effect of topical IOP-lowering medications on the TM and SC microstructure using EDI OCT.

## Methods

### Participants

This retrospective, observational study was approved by the Institutional Review Board of Korea University Ansan Hospital. It was conducted in accordance with the tenets of the Declaration of Helsinki. In this study, we further analyzed AS-OCT data from the previous study that investigated changes of anterior scleral thickness before and after the use of IOP-lowering medication in glaucoma patients^41^. Written informed consent was obtained from all subjects enrolled in the previous study.

Treatment-naïve patients with POAG who were first diagnosed at Korea University Ansan Hospital and were prescribed either PG analogues or DTFCs were eligible to be included herein. The drug selection was based on the judgement of a single physician (JHP) or on the patients’ preference (e.g., once-daily dosing regimen). Primary open-angle glaucoma was diagnosed when the subject showed glaucomatous optic disc change with reproducible glaucomatous VF defects and open angles on static gonioscopy. The following defects were identified as glaucomatous optic disc changes: (1) focal or diffuse neuroretinal rim thinning, (2) localized notching, or (3) RNFL defects. Glaucomatous VF defects were defined when two of the following three criteria were present: (1) a cluster of three or more non-edge contiguous points in the pattern deviation plot, with a < 5% probability of being present in age-matched healthy individuals (one of which was < 1%) without crossing the horizontal meridian; (2) pattern standard deviation of < 0.05; and (3) glaucoma hemifield test results outside normal limits.

Patients were excluded if they met one of the following criteria: (1) secondary glaucoma; (2) invisible TM in any quadrant on static gonioscopy; (3) previous ocular trauma or intraocular surgery, except uncomplicated cataract surgery more than 1 year before enrollment; (4) presence of retinal disease; (5) refractive error exceeding the spherical equivalent of 6 diopters or astigmatism of 3 diopters; (6) use of any type of topical eye drops, including topical IOP-lowering agents; or (7) unwillingness or inability to content to the study protocol.

All patients underwent ophthalmic examinations in both eyes at study enrollment, including best-corrected visual acuity assessment, refractive error measurement, slit lamp biomicroscopy examination, IOP measurement with Goldmann applanation tonometry, CCT evaluation using a non-contact specular microscope (SP-2000p; Topcon, Tokyo, Japan), axial length measurement using IOLMaster (Carl Zeiss Meditec, Jena, Germany), gonioscopy, Humphrey VF testing using the Swedish Interactive Threshold Algorithm 24–2 test (Zeiss-Humphrey, San Leandro, CA, USA), spectral-domain OCT, and dilated 30-degree stereoscopic photography and 50-degree red-free photography using a Zeiss FF 450 plus IR camera (Carl Zeiss Meditec Inc., Dublin, CA, USA). After usage of topical IOP-lowering agents for 3 months, slit lamp biomicroscopy examination, IOP measurement, CCT evaluation, and OCT examination were repeated in all subjects.

### EDI OCT

The spectral-domain OCT (Heidelberg Spectralis OCT, Heidelberg Engineering, Heidelberg, Germany) imaging was performed by a single experienced technician blinded to the patients’ glaucoma status and medication. The instrument utilizes a super luminescent diode with a central wavelength of 870 nm for OCT, and measurements were set to image a 20 × 5-degree rectangle in the sclera mode. Images of the nasal and temporal corneoscleral limbus were obtained with serial horizontal EDI B-scans. At each measurement session, the conjunctival vessels and iris anatomy were used as landmarks to scan the same limbal area before and after the usage of IOP-lowering agents. After acquisition of the before-and after-medication scans, the aqueous and blood vessels in each EDI B-scan were carefully reviewed to select one scan each and compared the same area before and after the usage of IOP-lowering agents.

### TM width, TM thickness, and SC area measurement

**Figure 2:**
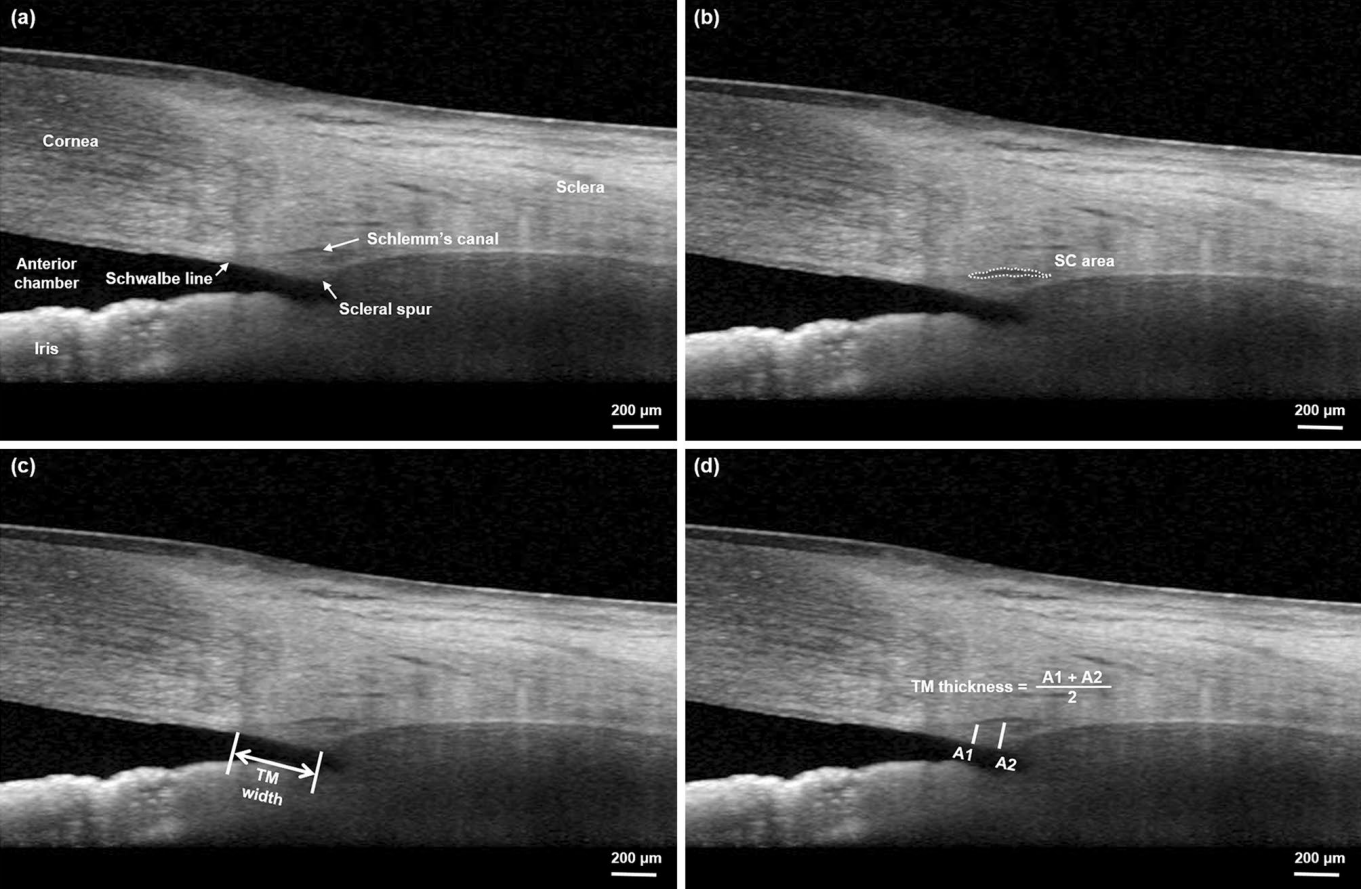
Measurements of the trabecular meshwork (TM) width, TM thickness, and Schlemm’s canal (SC) area. (**a**) The enhanced depth imaging optical coherence tomography B-scan was used to measure the parameters. (**b**) The black oval space was identified as SC and its area was measured by manually delineating the SC lumen. (**c**) The TM width was defined as the distance from the scleral spur to the Schwalbe line. (**d**) The thickness of TM was calculated as the average of two measurements, the perpendicular distances from the anterior endpoint (A1) and midpoint (A2) of SC to the inner layer of the TM.

Patients with an incomplete set of EDI OCT B-scans or with poor quality scans in which the TM and SC could not be reliably recognized were excluded from the analysis. The TM width, TM thickness, and SC area were measured by a single observer (HWC) who was blinded to the study protocol and timing of OCT (Fig. 2). The TM width was defined as the distance from the scleral spur to the Schwalbe line and was measured manually using the built-in caliper of the software provided in the OCT instrument. The location of the scleral spur was selected as the point where there was a change in the curvature of the inner surface of the angle wall, often presenting as an inward protrusion of the sclera^42,43^. The TM thickness was defined as beams and empty space and calculated as the average of two measurements, the perpendicular distances from the anterior endpoint and midpoint of SC to the inner layer of the TM^44,45^. The cross-sectional area of SC was measured by manually delineating the SC lumen using ImageJ (version 1.52, National Institutes of Health, Bethesda, MD, USA).

### Statistics

Statistical analysis was performed using SPSS (version 21.0; SPSS, Chicago, IL, USA). The normality of data distribution was verified using the Shapiro–Wilk normality test. The patients were divided into two groups according to the medications, and only one eye per subject was used for the analysis. If both eyes were eligible for inclusion in the study, we selected the eye randomly from each patient for analysis. For TM and SC microstructure measurements, the inter-observer reproducibility was assessed using the intraclass correlation coefficient in randomly selected 25 images of 25 eyes. The baseline characteristics, axial length, CCT, VF parameters, and IOP differences between the two groups were compared using either the independent t-test or the Mann–Whitney U test, as appropriate. The IOP, CCT, TM width, TM thickness, and SC area alterations before and after IOP-lowering medication usage were evaluated using the Wilcoxon signed-rank test. We performed Spearman’s correlation analysis to evaluate the relationship between the IOP reduction amount and changes in TM width, TM thickness, and SC area. *P* values of < 0.05 were considered statistically significant.

## Supplementary Information

Supplementary Tables.
