# Supplementary material for: Morphological changes in the trabecular meshwork and Schlemm’s canal after treatment with topical intraocular pressure-lowering agents
Source: Sci Rep. 2021 Sep 13;11:18169. doi: 10.1038/s41598-021-97746-x (PMC8437975; doi:10.1038/s41598-021-97746-x)
Supplement: Supplementary file 1 — Supplementary Tables. [file 41598_2021_97746_MOESM1_ESM.pdf]

Morphological Changes in the Trabecular Meshwork and Schlemm's Canal after Treatment with Topical Intraocular Pressure-Lowering Agents

Ji-Hye Park[1]*, Hyun Woo Chung[1], Eun Gyu Yoon[1], Min Jung Ji[2], Chungkwon Yoo[1], Yong Yeon Kim[1]

1. Department of Ophthalmology, Korea University College of Medicine, Seoul, South Korea

2. Seoul Best Eye Clinic, Seoul, South Korea

Corresponding author:

Ji-Hye Park, MD, PhD

Department of Ophthalmology, Korea University Ansan Hospital,

123 Jeokgeum-ro, Danwon-gu, Ansan-si, Gyeonggi-do 15355, South Korea

E-mail: jennypark8321@gmail.com

Tel: 82-31-412-5160

Fax: 82-31-412-4267

Supplementary Table 1. Intraclass Correlation Coefficient (ICC) of the Measurements of Trabecular Meshwork (TM) and Schlemm's Canal (SC) Microstructure

	ICC	95% CI
TM width		
Nasal sector	0.937	0.858-0.972
Temporal sector	0.920	0.818-0.965
TM thickness		
Nasal sector	0.976	0.945-0.989
Temporal sector	0.982	0.958-0.992
SC area		
Nasal sector	0.926	0.832-0.967
Temporal sector	0.912	0.800-0.961

Supplementary Table 2. Comparison between Two Prostaglandin Subgroups According to the Amount of Intraocular Pressure (IOP) Reduction

	Small IOP reduction group (n = 12)	Large IOP reduction group (n = 21)	*P* value*
Baseline IOP (mmHg)	15.1 ± 1.7	19.0 ± 3.2	< 0.001
CCT	532.42 ± 31.15	527.95 ± 38.87	0.699
AXL	24.34 ± 0.87	24.52 ± 1.51	0.674†
MD (dB)	-4.42 ± 3.68	-5.23 ± 4.68	0.567
RNFL thickness	84.83 ± 12.14	84.14 ± 13.47	0.884†
TM width			
Nasal sector	529.91 ± 91.09	510.40 ± 66.05	0.792
Temporal sector	491.67 ± 59.41	504.53 ± 63.53	0.704
TM thickness			
Nasal sector	86.86 ± 12.99	88.64 ± 15.64	0.966
Temporal sector	88.64 ± 11.34	91.44 ± 18.09	0.826
SC area			
Nasal sector	4308.94 ± 942.71	5199.61 ± 1536.87	0.104
Temporal sector	4517.38 ± 1590.29	4758.35 ± 1681.40	0.675
△IOP	-1.3 ± 1.4	-5.5 ± 1.6	< 0.001
△CCT	-3.00 ± 5.61	-11.95 ± 9.21	0.004
△TM width			
Nasal sector	-14.82 ± 24.29	4.80 ± 45.96	0.104
Temporal sector	-3.25 ± 30.48	5.16 ± 31.59	0.220
△TM thickness			
Nasal sector	5.33 ± 12.32	13.85 ± 13.02	0.027
Temporal sector	5.90 ± 15.40	5.63 ± 12.20	0.659
△SC area			
Nasal sector	-403.19 ± 905.36	-652.41 ± 918.08	0.761
Temporal sector	-352.05 ± 645.20	-737.96 ± 855.66	0.164

*Mann–Whitney U test, †Independent t-test

IOP, intraocular pressure; CCT, central corneal thickness; AXL, axial length; MD, mean deviation; RNFL, retinal nerve fiber layer; TM, trabecular meshwork; SC, Schlemm’s canal
